# Accuracy of upper endoscopies with random biopsies to identify patients with gastric premalignant lesions who can safely be exempt from surveillance

**DOI:** 10.1007/s10120-020-01149-2

**Published:** 2021-02-22

**Authors:** Michiel C. Mommersteeg, Stella A. V. Nieuwenburg, Wouter J. den Hollander, Lisanne Holster, Caroline M. den Hoed, Lisette G. Capelle, Tjon J. Tang, Marie- Paule Anten, Ingrid Prytz-Berset, Ellen M. Witteman, Frank ter Borg, Jordy P. W. Burger, Michail Doukas, Marco J. Bruno, Maikel P. Peppelenbosch, Gwenny M. Fuhler, Ernst J. Kuipers, Manon C. W. Spaander

**Affiliations:** 1 Department of Gastroenterology and Hepatology, Erasmus University Medical Centre, Rotterdam, The Netherlands; 2 Department of Gastroentroenterology and Hepatology, Leiden University Medical Centre, Leiden, The Netherlands; 3 Department of Gastroentroenterology and Hepatology, Meander Medical Centre, Amersfoort, The Netherlands; 4 Department of Gastroentroenterology and Hepatology, IJsselland Hospital, Capelle Aan Den IJssel, The Netherlands; 5 Department of Gastroentroenterology and Hepatology, Sint Franciscus Hospital, Rotterdam, The Netherlands; 6 Department of Gastroentroenterology and Hepatology, More and Romsdal Trust Ålesund, Ålesund, Norway; 7 Department of Gastroentroenterology and Hepatology, Canisius-Wilhelmina Hospital, Nijmegen, The Netherlands; 8 Department of Gastroentroenterology and Hepatology, Deventer Hospital, Deventer, The Netherlands; 9 Department of Gastroenterology and Hepatology, Rijnstate, Arnhem, The Netherlands; 10 Department of Pathology, Erasmus University Medical Centre, Rotterdam, The Netherlands

**Keywords:** Surveillance, Prevention, Gastric cancer, Atrophic gastritis, Intestinal metaplasia

## Abstract

**Introduction:**

Guidelines recommend endoscopy with biopsies to stratify patients with gastric premalignant lesions (GPL) to high and low progression risk. High-risk patients are recommended to undergo surveillance. We aimed to assess the accuracy of guideline recommendations to identify low-risk patients, who can safely be discharged from surveillance.

**Methods:**

This study includes patients with GPL. Patients underwent at least two endoscopies with an interval of 1–6 years. Patients were defined ‘low risk’ if they fulfilled requirements for discharge, and ‘high risk’ if they fulfilled requirements for surveillance, according to European guidelines (MAPS-2012, updated MAPS-2019, BSG). Patients defined ‘low risk’ with progression of disease during follow-up (FU) were considered ‘misclassified’ as low risk.

**Results:**

334 patients (median age 60 years IQR11; 48.7% male) were included and followed for a median of 48 months. At baseline, 181/334 (54%) patients were defined low risk. Of these, 32.6% were ‘misclassified’, showing progression of disease during FU. If MAPS-2019 were followed, 169/334 (51%) patients were defined low risk, of which 32.5% were ‘misclassified’. If BSG were followed, 174/334 (51%) patients were defined low risk, of which 32.2% were ‘misclassified’. Seven patients developed gastric cancer (GC) or dysplasia, four patients were ‘misclassified’ based on MAPS-2012 and three on MAPS-2019 and BSG. By performing one additional endoscopy 72.9% (95% CI 62.4–83.3) of high-risk patients and all patients who developed GC or dysplasia were identified.

**Conclusion:**

One-third of patients that would have been discharged from GC surveillance, appeared to be ‘misclassified’ as low risk. One additional endoscopy will reduce this risk by 70%.

**Supplementary Information:**

The online version contains supplementary material available at 10.1007/s10120-020-01149-2.

## Introduction

Prognosis of advanced gastric cancer is poor, with a five-year-survival rate of 20% [1]. However, if gastric cancer is detected at an early stage, survival rates improve up to 90% [2, 3]. Chronic atrophic gastritis (CAG) and intestinal metaplasia (IM) are precursor lesions of gastric adenocarcinoma (GC) [4]. These premalignant lesions make gastric cancer suitable for screening and surveillance, depending on the regional prevalence of (pre-)malignant lesions and progression rates to cancer. Nationwide screening strategies are mainly relevant for high-GC prevalence regions such as East-Asia, where population-based screening programs are implemented [5]. In case advanced premalignant lesions are detected, patients are eligible for surveillance and early intervention when possible [6]. For regions with low prevalence rates, such as Western Europe and North America, nationwide screening is not recommended due to a low a priori risk. However, when patients of these regions are inadvertently diagnosed with advanced premalignant lesions, for instance, during routine endoscopy, surveillance should be considered given the risk of progression to cancer. The debate about the balance between harms and benefits of surveillance strategies is still ongoing [7]. To this end, US guidelines state that surveillance is not indicated except for individuals with a known increased gastric cancer risk, such as persons of Asian ancestry or patients with a positive family history [8]. European MAPS (management of epithelial precancerous conditions and lesions in the stomach) as well as the British guidelines, recommend surveillance in patients with a premalignant gastric lesion [9–11]. These surveillance recommendations depend on the extent and severity of these premalignant lesions. The MAPS-2012 guideline recommended surveillance for patients with extensive CAG or IM. In the updated MAPS-2019 guideline, surveillance was extended to patients with CAG and IM limited to either the antrum or the corpus and presence of incomplete intestinal metaplasia (in any of the biopsies), autoimmune gastritis, persistent *Helicobacter pylori (H. pylori)* infection or a first degree relative with gastric cancer. In 2019, the British Society of Gastroenterology also published a guideline (BSG) on the management of gastric premalignant lesions. The recommendations on surveillance of premalignant lesions were mostly identical to the revised MAPS-2019 guideline. The only difference with the MAPS guideline is that patients with autoimmune gastritis and CAG or IM limited to the antrum or corpus were not referred for further surveillance.

To assess the extent and severity of premalignant gastric lesions, endoscopic surveillance is advised. However, endoscopic recognition of gastric premalignant lesions can be difficult. Therefore, obtaining random biopsies throughout the stomach according to the updated Sydney protocol is recommended [12]. Nevertheless, due to the uneven distribution of gastric premalignant lesions, random biopsies may not properly reflect the extent of the lesions and subsequently the individual gastric cancer risk [13]. Since only a few patients will develop gastric cancer, it is essential that surveillance strategies in practice lead to the identification of those few cases. While the addition of new risk factors to the MAPS-2019 and BSG potentially allows better stratification of at-risk patients, thus far, there is only limited data describing to what extent we can accurately dismiss patients with premalignant gastric lesions from surveillance, based on their low risk of gastric cancer development. The aim of this study was to assess to what extent we can accurately identify low-risk patients who can safely be discharged from gastric surveillance according to the recommendations of MAPS-2012, MAPS-2019 and BSG guideline.

## Methods

### Study design

This study is based on the Proregal study (Progression and Regression of precancerous Gastric Lesions). The design of the study has been described previously [14]. In short, this study was initiated in 2009 and is an ongoing prospective cohort study carried out in six hospitals (one academic, five regional) in the Netherlands and one regional hospital in Norway. Patients are eligible for inclusion if they are over 18 years of age and diagnosed with one of the following conditions at routine endoscopy (t0): atrophic gastritis, intestinal metaplasia and/or dysplasia in any part of the gastric mucosa. Patients are excluded from participation if they have: (1) previously undergone upper gastrointestinal surgery, (2) a previous diagnosis of gastric carcinoma, or any other malignancy not being in remission, (3) severe comorbidity limiting their expected survival to less than 2 years, (4) portal hypertension, or (5) a proven *CDH1* mutation. In case *H. pylori* *was present,* *H. pylori* eradication was provided, and eradication was verified. in all patients (20 patients had persistent *H. pylori* colonization). *H. pylori* eradication was eventually achieved in all patients.

**Fig. 1:**
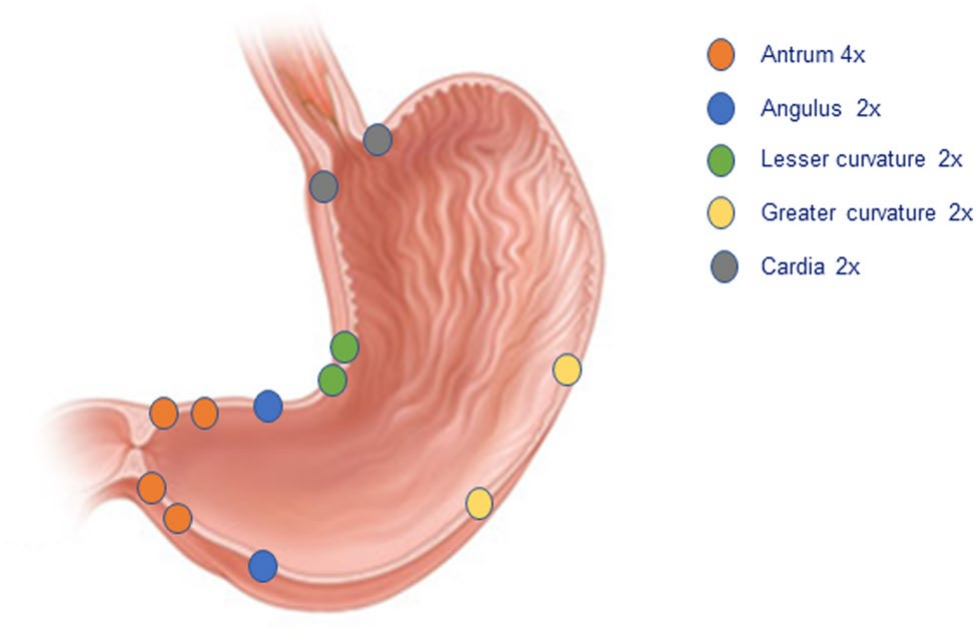
Locations of standardised random gastric biopsies obtained during endoscopy for this study

In all subjects, a surveillance endoscopy is performed at one (t1) and three (t2) years after the initial endoscopy. Random biopsy samples are obtained according to the Proregal biopsy protocol with targeted biopsies in case of visible gastric lesions (Fig. 1).

In case of low-grade dysplasia (LGD) or high-grade dysplasia (HGD), the surveillance interval is shortened to twelve and six months, respectively. In case a visible lesion is detected, endoscopic resection of the lesion is performed. After t2, continuation or cessation of surveillance is decided based on the recommendations of the MAPS guideline. For the purpose of this study, subjects who were discharged from further surveillance based on the MAPS guideline recommendation at t2 were re-invited for endoscopy (t3).

### Patient selection

All patients from the Proregal cohort were eligible for inclusion provided patients had at least one follow-up endoscopy. Patients were classified as low or high risk. Low-risk patients were defined as patients with premalignant gastric lesions, who do not fulfil the requirements for surveillance (i.e. IM limited to the antrum). ‘High risk’ patients are patients with premalignant gastric lesions who are advised to undergo surveillance (i.e. IM in both antrum and corpus). Requirements for surveillance are described in more detail in Supplementary Table 1.

At baseline, patients were defined as ‘low risk’ for progression of disease if they fulfilled requirements for discharge based on the European guidelines MAPS-2012, MAPS-2019 or the BSG guideline. Patients were defined as ‘high risk’ if further surveillance was indicated according to these guidelines.

To assess the safety of discharging patients from further surveillance based on MAPS/BSG guideline recommendations, we included the ‘low risk’ patients who had no indication for further surveillance according to the guidelines at t1 or t2 (e.g. limited extension of IM), but underwent a surveillance endoscopy within the scope of this study. We correlated the findings with the outcome of the endoscopy performed at t2 and t3, respectively. In case lesions were found at the follow-up endoscopy for which surveillance is recommended, the patient was defined as ‘misclassified’ as low risk for gastric cancer development.

**Fig. 2:**
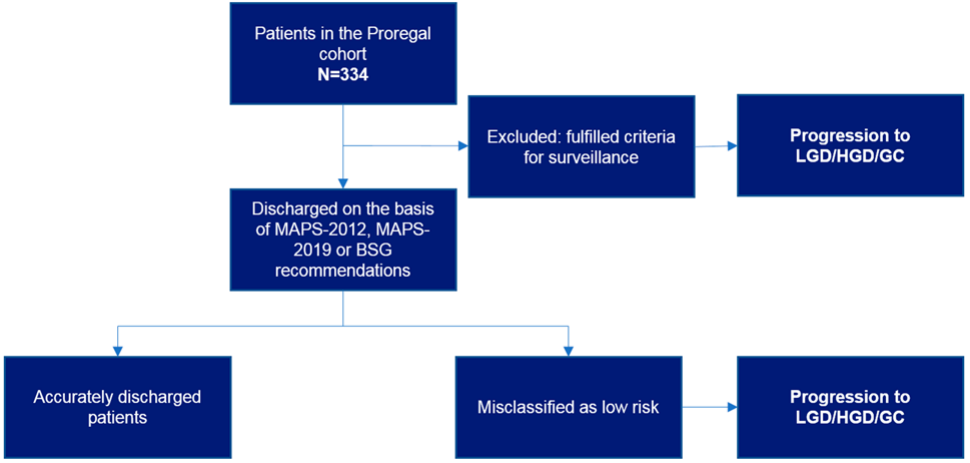
Flowchart of the study design. *GC* gastric cancer; *HGD* high-grade dysplasia; *LGD* low-grade dysplasia; *MAPS* management of precancerous conditions and lesions in the stomach; *Proregal* progression and regression of precancerous lesions

Furthermore, we linked all patients to the Netherlands Cancer Registry, managed by the Netherlands Comprehensive Cancer Organisation (IKNL) to account for all (interval) gastric cancers even after surveillance was stopped. Since 1989, the Netherlands Cancer Registry registers all participants diagnosed with cancer in the Netherlands. The study design is depicted in Fig. 2. The study protocol was approved by the Institutional Review Board (MEC-2009-090).

### MAPS-2012

The MAPS guideline recommendations were first published in 2012 [9]. Chronic atrophic gastritis (CAG) or intestinal metaplasia (IM) confined to the antrum did not require further surveillance. CAG or IM in both antrum and corpus or only in the corpus required endoscopic surveillance every three years (Suppl. Table 1).

### MAPS-2019

The MAPS guideline recommendations were updated in 2019 [10]. CAG or IM limited to the antrum or corpus does not require further surveillance. However, surveillance every three years is recommended if the subject meets one of the following criteria: first degree family history of gastric cancer, autoimmune gastritis, persistent *H. pylori* infection or incomplete IM (Table 1). Surveillance every 1–2 years is recommended in case of a first degree relative with GC (Suppl. Table 1).

**Table 1:** Baseline characteristics of the total Proregal cohort

	Proregal cohort (*n* = 334)
Gender (male, %)	48.7
Age (median, IQR)	60 (11)
*H. pylori* positive (histology)	103 (31)
Follow-up months (median, IQR)	48 (24)
Most severe lesion recent endoscopy^a^	
OLGIM 0	123 (36)
OLGIM I	55 (16)
OLGIM II	81 (24)
OLGIM III	51 (15)
OLGIM IV	9 (3)
Lesions requiring treatment	
LGD	1
HGD	2
GC	4
High-risk features	
Persistent *H. pylori* infection	20
Autoimmune gastritis	20
First degree family history of GC	47

*H. pylori Helicobacter pylori*; *IQR* inter quartile range; *GC* gastric cancer; *HGD* high-grade dysplasia; *LGD* low-grade dysplasia; *OLGIM* operative link on gastric intestinal metaplasia assessment; *Proregal cohort* total number of the prospective cohort that are or have been under surveillance for their gastric premalignant lesions

^a^Capelle et al*., Gastrointest Endosc., 2010* [31]

### BSG

The BSG guideline recommendations were published in 2019 [11]. In this guideline, CAG or IM limited to the antrum or corpus does not require further surveillance. However, surveillance every three years is recommended if the subject meets one of the following criteria: first degree family history of gastric cancer or persistent *H. pylori* infection (Table 1).

### Statistical analyses

Baseline characteristics are presented as mean with standard deviation (SD) or median with interquartile range (IQR) when appropriate. Kaplan–Meier curves were performed on the proportion of patients identified as still at risk after (supposed) discharge of surveillance per year and per endoscopy for MAPS-2012, MAPS-2019 and BSG guidelines.The analyses were performed using SPSS v.24 statistical package (SPSS Inc, Chicago, IL).

## Results

### Patient characteristics

In total, 334 patients were included. Median age was 60 years (IQR 11) and 48.7% were men. This cohort captured seven cases of dysplasia or gastric cancer. Baseline characteristics of the entire cohort are shown in Table 1 and were described in more detail previously [14].

### Following the MAPS-2012 guideline recommendation

**Fig. 3:**
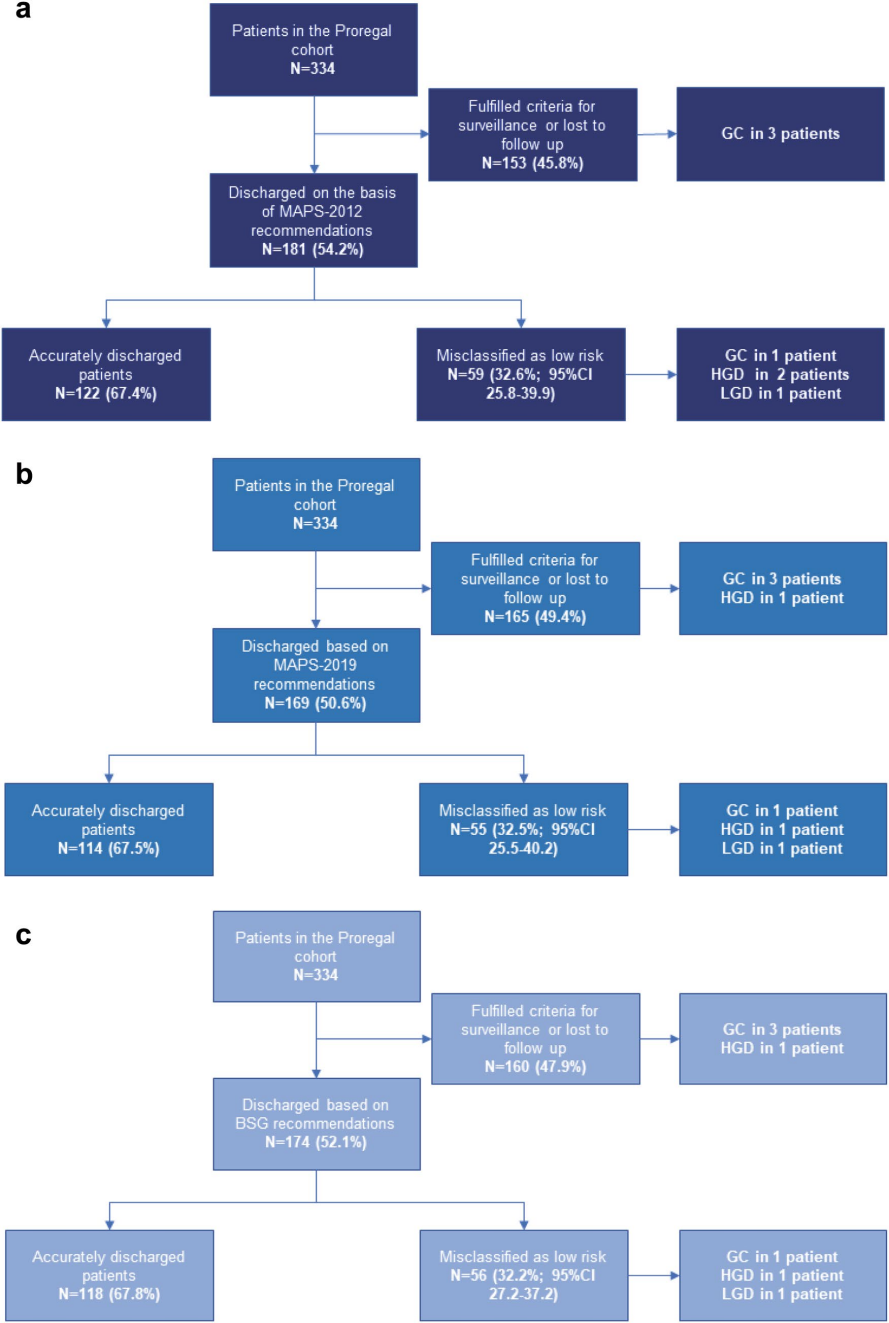
Schematic visualization of surveillance of patients with premalignant gastric lesion according to the MAPS-2012 guideline (**a**), according to the MAPS-2019 guideline (**b**) and according to the BSG (**c**). *LGD* low-grade dysplasia; *HGD* high-grade dysplasia; *GC* gastric cancer; *CI* confidence interval; *MAPS* management of precancerous conditions and lesions in the stomach; *Proregal* progression and regression of precancerous lesions

At baseline, 153/334 (45.8%) patients were correctly defined as high risk (i.e. already fulfilled the criteria for surveillance according to the guideline) and therefore excluded. Of the remaining 181 low-risk patients (i.e. would have been discharged from further surveillance according to the guideline), 59 patients (32.6%) were misclassified as low risk because they had gastric lesions at subsequent endoscopies that gave reason to continue surveillance (i.e. gastric premalignant lesions not limited to the antrum) see also Fig. 3a. This included four out of the total seven cases of LGD/HGD/gastric cancer cases. LGD was found in one patient, HGD was found in two patients, and one patient developed gastric cancer. One of the patients with HGD underwent endoscopic resection and died from causes unrelated to gastric cancer or the procedure. The other patient with HGD underwent a successful gastrectomy. The patient with gastric cancer and the patient with LGD underwent an endoscopic resection and are currently in remission for over two years. Patient characteristics of these cases are available in Table 2.

**Table 2:** Characteristics of patients identified with a resectable lesion in the complete proregal cohort including which guideline would have accurately identified these patients being at risk

#	Age diagnosis	Gender	First diagnosis of GPL	Guideline recommending surveillance	Lesions at end of surveillance	High-risk feature(s)	OLGIM (most severe)	Lesion found in follow-up	Therapy
1	78^a^	Male	1996	None	Moderate intestinal metaplasia of antrum and angulus	None	III	High-grade dysplasia antrum	Endoscopic resection
2	57	Male	2010	None	Moderate intestinal metaplasia of antrum and angulus	None	IV	Intestinal-type adenocarcinoma angulus	Endoscopic resection
3	64	Female	2009	None	Slight intestinal metaplasia of the antrum	None	III	Low-grade dysplasia of the antrum	Endoscopic resection
4	77	Male	1996	MAPS-2019 BSG	Chronic gastritis of antrum and corpus	First degree family with GC, AI gastritis	IV	High-grade dysplasia of the antrum	No therapy
5	46	Male	2008	MAPS-2019 MAPS-2012 BSG	N/A	First degree family with GC	II	Intestinal-type adenocarcinoma antrum	Endoscopic resection
6	53	Female	2009	MAPS-2012 MAPS-2019 BSG	N/A	First degree family with GC	III	Diffuse-type gastric cancer	Total gastrectomy
7	72^a^	Male	2006	MAPS-2012 MAPS-2019 BSG	N/A	None	IV	Intestinal-type adenocarcinoma Lesser curvature/angulus	Total gastrectomy

*GPL* gastric premalignant lesion; *MAPS* management of precancerous conditions and lesions in the stomach; *BSG* British society of Gastroenterology guideline; *N/A* not applicable; *GC* gastric cancer; *AI* autoimmune

^a^Patient is deceased

### Following the MAPS-2019 guideline recommendation

At baseline, 165/334 (49.4%) patients were correctly defined as high risk (i.e. already fulfilled the criteria for surveillance according to the guideline) and therefore excluded. Of the remaining 169 low-risk patients (i.e. would have been discharged from further surveillance according to the guideline), 55 patients (32.5%) were misclassified as low risk because they had gastric lesions at subsequent endoscopies that gave reason to continue surveillance (i.e. gastric premalignant lesions not limited to the antrum). This included three out of the total seven cases of LGD/HGD/gastric cancer (one case of LGD, HGD and gastric cancer each) see also Fig. 3b. These patients were also misclassified by the MAPS-2012 guideline. The correctly classified patient with HGD (that was misclassified in MAPS-2012) was included for surveillance according to MAPS-2019 because of a first degree relative with gastric cancer. This patient had HGD, but declined further therapy due to comorbidities and age, although (endoscopic) resection might have been possible. Another patient with carcinoma who would have been discharged according to the extent of his lesions (limited to the corpus) was recommended to undergo surveillance according to the MAPS-2019 guideline, also because of a first degree relative with gastric cancer. Patient characteristics of these cases are depicted in Table 2.

### Following the BSG guideline recommendation

At baseline, 160/334 (47.9%) patients were correctly defined as high risk (i.e. already fulfilled the criteria for surveillance according to the guideline) and therefore excluded. Of the remaining 174 low-risk patients (i.e. would have been discharged from further surveillance according to the guideline), 56 patients (32.2%) were misclassified as low risk because they had gastric lesions at subsequent endoscopies that gave reason to continue surveillance (i.e. gastric premalignant lesions not limited to the antrum). This included three out of the total seven cases of LGD/HGD/gastric cancer (one case of LGD, HGD and gastric cancer each) see also Fig. 3c. These patients were also misclassified by the MAPS-2012 and MAPS-2019 guideline. The correctly classified HGD patient (who was misclassified in MAPS-2012) was included for surveillance according to BSG because of a first degree relative with gastric cancer. This patient had HGD, but declined further therapy due to comorbidities and age, although (endoscopic) resection might have been possible. Another patient with carcinoma who would have been discharged according to the extent of his lesions (limited to the corpus) was recommended to undergo surveillance according to the BSG guideline, also because of a first degree relative with gastric cancer. Patient characteristics of these cases are depicted in Table 2.

### Longitudinal follow-up

**Fig. 4:**
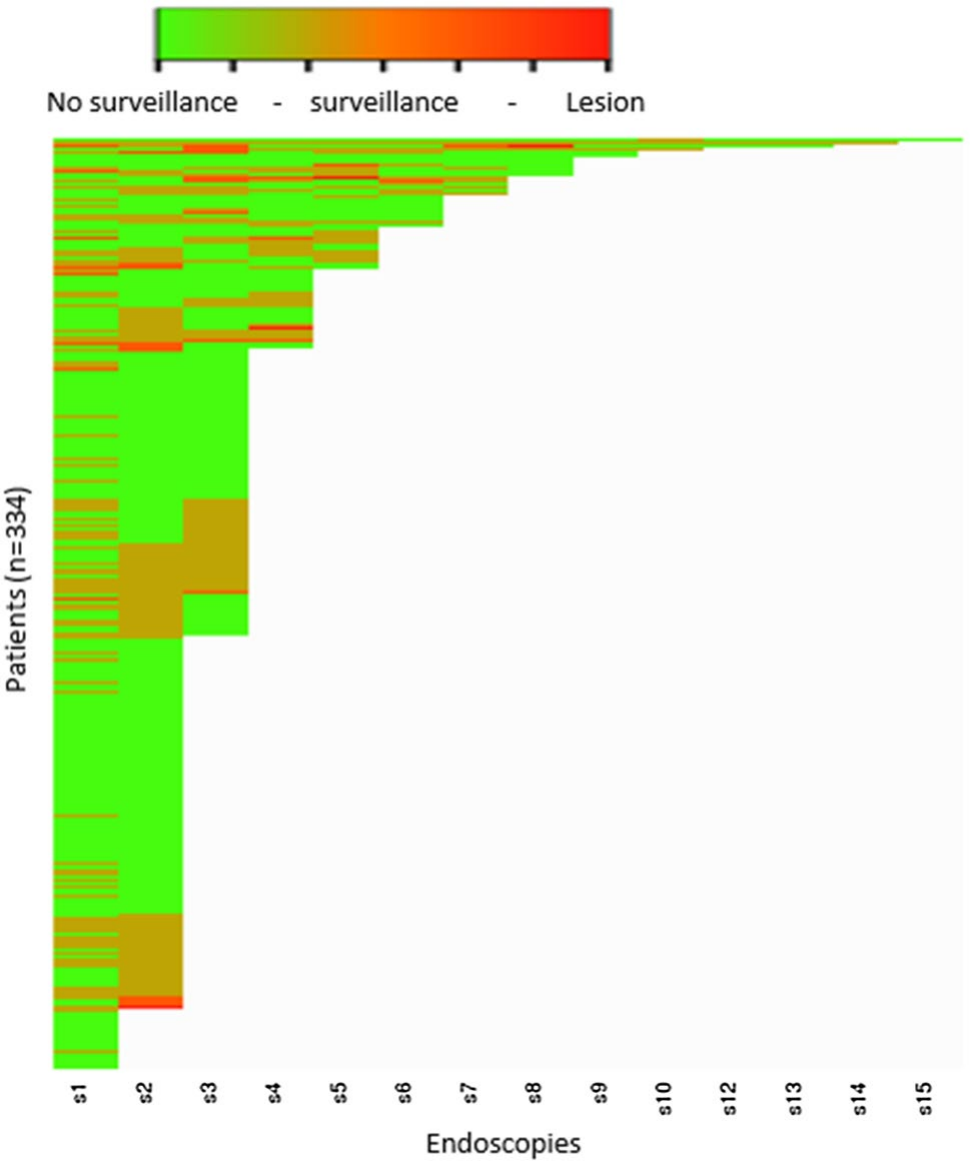
Heat map of the outcomes of the PROREGAL study, based on MAPS-2019 guideline. On the *Y*-axis, all individual patients have been plotted, on the *X*-axis, the endoscopies are shown. A *green colour* indicates that MAPS-2019 would not recommend an endoscopy, an *orange colour* represents a recommendation of surveillance. A *red colour* represents detection of lesions that should be considered for (endoscopic) resection (i.e. LGD, HGD, gastric adenocarcinoma). Several patients who would have been discharged based on the lesion found and according to MAPS-2019 guideline did show lesions warranting surveillance in subsequent endoscopies

**Fig. 5:**
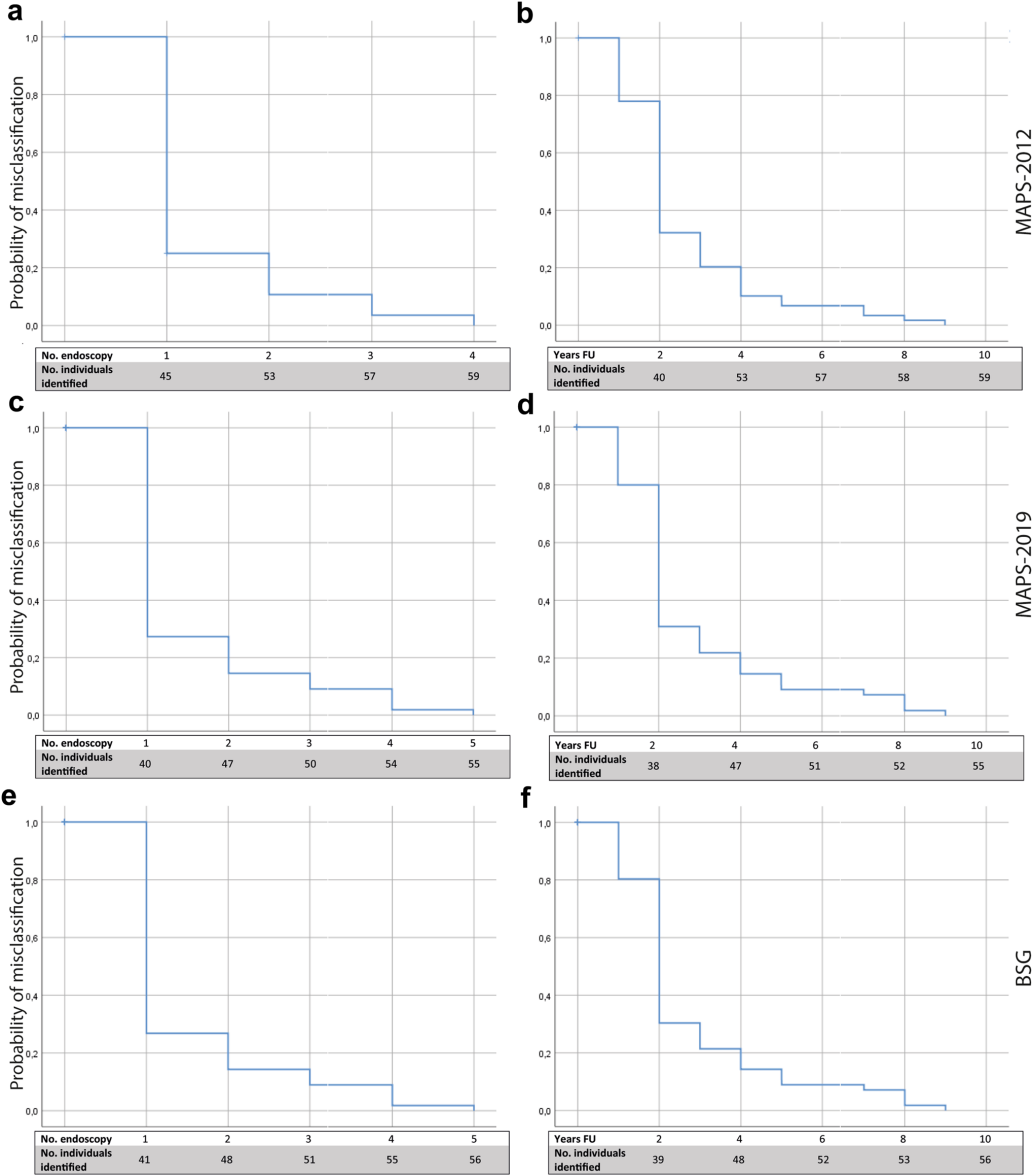
Kaplan–Meier analysis of the probability of patients being misclassified as low risk for gastric cancer. **a** Patients who were misclassified as low risk according to MAPS-2012, distinguished by number of endoscopies needed until identified as still at risk. **b** Patients who were misclassified as low risk according to MAPS-2012, distinguished by years of follow-up until identified as still at risk. **c** Patients who were misclassified as low risk according to MAPS-2019, distinguished by number of endoscopies needed until identified as still at risk. **d** Patients who were misclassified as low risk according to MAPS-2019, distinguished by years of follow-up until identified as still at risk. **e** Patients who were misclassified as low risk according to BSG, distinguished by number of endoscopies needed until identified as still at risk. **f** Patients who were misclassified as low risk according to BSG, distinguished by years of follow-up until identified as still at risk. *No.* number of; *FU* follow-up; *MAPS* management of precancerous conditions and lesions in the stomach

Figure 4 shows a graphical presentation of the results of each endoscopy according to the MAPS-2019 guideline. As can be appreciated from this figure, several patients continuously switch between high and low risk of progression. The number of endoscopies or time necessary to correctly identify low-risk patients was visualized by a Kaplan–Meier curve. As seen in Fig. 5a, c, e, one additional endoscopy identifies over 75.4% (95% CI 64.3–86.6), 72.9% (95% CI 62.4–83.3) and 73.2% (95% CI 59.7–84.2) of high-risk patients according to the MAPS-2012, MAPS-2019 or BSG guideline, respectively. After two additional endoscopies, this percentage increases to 89.5% (95% CI 81.5–97.5), 85.7% (95% CI 77.5–93.9) and 85.7 (95% CI 73.8–93.6 or BSG, respectively. Figure 5b, d, f shows patients who were defined as low risk according to MAPS-2012, MAPS-2019 or BSG by years of follow-up. After 3 years, the percentage of correctly identified patients at low gastric cancer development risk is 77.2% (95% CI 66.3–88.1), 80.9% (95% CI 74.1–91.7) and 80.4% (95% CI 67.6–89.8) as shown in Fig. 5b, d, f. In this period, all patients underwent either one or two additional endoscopies. All of the misclassified low-risk patients, who were diagnosed with GC or dysplasia during follow-up, would have been correctly classified as high risk in case one additional endoscopy between two and four years was performed (following any guideline).

## Discussion

To the best of our knowledge, this is the first study that evaluates how accurate we can identify low-risk GPL patients, and whether they can be safely discharged from further surveillance based on guideline recommendations. Due to the addition of several risk factors in MAPS-2019 and BSG as compared to MAPS-2012, MAPS-2019 and BSG improve the identification of high-risk patients who develop lesions requiring treatment, while reducing the amount of patients under surveillance. Nevertheless, a large proportion of patients at risk of neoplastic progression are still missed. Up to 32% of patients who are discharged from gastric cancer surveillance appeared to be misclassified as ‘low risk’ on subsequent endoscopy, including three out of the total of seven HGD/GC cases following the current European guidelines (MAPS-2019 and BSG). By adding one additional endoscopy, all patients who developed dysplasia or cancer were correctly identified as high risk and risk of misclassification was reduced by 72.9%. Dismissing patients from further surveillance after two ‘negative’ endoscopies is already common practice in colonic polyp surveillance and might be considered in the setting of gastric premalignant lesions [15].

Currently, extension and severity of gastric premalignant lesions are determined by upper endoscopy with random biopsies. This might cause sampling error and with that, underestimation of the true extent of lesions [16, 17]. This could explain the number of patients who were misclassified as low risk in our study, based on the subsequent endoscopy results. It has been described previously that conventional white light endoscopy (WLE) findings do not correlate well to histological findings in gastric premalignant lesions [18, 19]. Advanced endoscopic techniques such as high-definition endoscopes and imaging enhancement technologies have improved markedly in the past years [20, 21]. High-Definition-White Light Endoscopy (HD-WLE) has improved the correlation between endoscopic and histological diagnosis, with a sensitivity ranging between 75% and 92.7% and a specificity ranging between 92.7% and 100% [20, 21]. However, when measured in daily clinical practice, this accuracy decreases to 53% sensitivity and 98% specificity [22]. The addition of Narrow band imaging (NBI) to high-resolution endoscopy can further enhance accuracy up to a sensitivity of 85% and a specificity of 77% [23]. Our study highlights the importance of embracing and further investigating endoscopic improvement tools to stratify at-risk individuals with better accuracy and make surveillance guidelines more efficient [23]. These improvements in endoscopic techniques opens possibilities for targeted biopsies during surveillance endoscopies, which is expected to lower sampling error. On the other hand, improvements in endoscopic techniques will only lead to a decrease in sampling error if endoscopist is trained to correctly interpret endoscopic images and identify the gastric lesions. MAPS-2019 guidelines recommend the use of NBI or chromoendoscopy as it outperforms the use of WLE alone as described above. However, the use of other virtual chromoendoscopy such as i-Scan or FICE is less well investigated. These new developments may contribute to a better detection of premalignant gastric lesions, but therefore further research is necessary, especially to assess the usability and yield in daily practice in non-expert hands before recommendations can be made.

Another potential explanation for the apparent changes in perceived severity of lesions during follow-up is that it represents regression of lesions, rather than sampling error. *H. pylori* eradication effectively blocks progression of intestinal metaplasia and reduces the risk of gastric cancer [24]. While regression of (chronic) atrophic gastritis has been suggested [25–27], actual regression or disappearance of IM has only been described in several individual studies. Indeed, several meta-analyses show no significant regression of IM even after *H. pylori* eradication and long-term treatment with a Cox2 inhibitor [28, 29]. This held true for premalignant lesions in all the different locations of the stomach and supports the idea that sampling error contributes more to the fluctuating severity of lesions in our cohort than biological pro- or regression of disease [29, 30]. This raises the question whether histology from the last available endoscopy only is sufficient to identify patients who are at risk for gastric cancer development. According to our data, performing one additional endoscopy will identify the majority of patients who otherwise would have been inappropriately discharged. A surveillance strategy in which longitudinal data of all previous endoscopies are taken into account in risk assessment should be a future step.

The main challenge of surveillance strategies, especially in low-risk areas, is to identify the small proportion of patients that will benefit from surveillance while circumventing the burdens of such strategy. No surveillance strategy will perfectly identify all patients at risk. Therefore, it is important to take into account the risk of progression of premalignant lesions, the availability of a relatively safe and effective method to treat (early) cancers and the burden due to surveillance for both patient as well as health care. In three (43%) out of the seven patients who developed gastric cancer or dysplasia, at least one high-risk feature was present. Our study shows that the addition of risk factors (autoimmune gastritis, first degree family history and persistent *H. pylori* infection) in MAPS-2019 and BSG indeed increases the yield of the current surveillance strategy and highlights the importance of continuing this improvement of risk stratification.

Several limitations have to be taken into account. First, this is an observational study which is not powered to make specific recommendations to improve current guidelines. This study solely provides descriptive information. Larger sample size in a randomized controlled trial setting would be needed to truly investigate the risk of misclassification and improve on guideline recommendations regarding biopsy strategies and surveillance (intervals). Furthermore, misclassification of patients is unavoidable and what percentage is deemed acceptable is debatable. This will depend on the costs health systems are willing to bear. Also, even though (almost) half of the dysplasia/gastric cancer cases in our cohort would never have received a surveillance endoscopy if guidelines were followed, these patients may have presented themselves when complaints would have arisen. Inevitably, this would have caused some delay in their treatment. However, the possibility of endoscopic treatment and outcome can only be speculated upon.

In conclusion, cancer detection improved with the updated MAPS-2019 and BSG guidelines, however, still three out of seven dysplasia/GC cases were missed. Furthermore, one-third of patients were misclassified as low risk for gastric cancer development and therefore would have inappropriately been discharged from further surveillance. The majority of these patients could be identified by performing one additional endoscopy within three years. Our study emphasizes the need for further improvement of stratifying at-risk individuals and improved endoscopic recognition of premalignant gastric lesions.

## Supplementary Information

Below is the link to the electronic supplementary material.

Supplementary file1 (DOCX 15 KB)

## Abbreviations

CAG: Chronic atrophic gastritis
GC: Gastric cancer
GPL: Gastric premalignant lesions
HGD: High-grade dysplasia
IM: Intestinal metaplasia
LGD: Low-grade dysplasia
MAPS: Management of precancerous conditions and lesions in the stomach
BSG: British society of gastroenterology
OLGIM: Operative link on gastric intestinal metaplasia
