# Supplementary material for: Accuracy of upper endoscopies with random biopsies to identify patients with gastric premalignant lesions who can safely be exempt from surveillance
Source: Gastric Cancer. 2021 Feb 22;24(3):680–90. doi: 10.1007/s10120-020-01149-2 (PMC8065002; doi:10.1007/s10120-020-01149-2)
Supplement: Supplementary file 1 — Supplementary file1 (DOCX 15 KB) [file 10120_2020_1149_MOESM1_ESM.docx]

**Supplementary files**

|  | MAPS-2012 | MAPS-2019 | BSG |
| --- | --- | --- | --- |
|  | **Surveillance (yes/no)** | **Surveillance (yes/no)** | **Surveillance (yes/no)** |
| CAG/IM antrum | No | Yes: every 3 years, if:   - Family history of GC - Incomplete IM - AIG - Persistent Hp infection   Absence of above = no surveillance | Yes: every 3 years, if:   - Family history of GC - Persistent Hp infection   Absence of above = no surveillance |
| CAG/IM corpus | Yes: every 3 years | Yes: every 3 years, if:   - Family history of GC - Incomplete IM - AIG - Persistent Hp infection   Absence of above = no surveillance | Yes: every 3 years, if:   - Family history of GC - Persistent Hp infection   Absence of above = no surveillance |
| CAG/IM antrum + corpus | Yes: every 3 years | Yes: every 1-2 years, if:   - First degree relative with GC   Absence of above = every 3 years | Yes: every 3 years |

**Table 1** Recommended management of premalignant stomach lesions according to the MAPS guideline 2012, the updated MAPS guideline 2019 and BSG guideline; *AIG; auto immune gastritis, CAG; chronic atrophic gastritis, GC; gastric cancer, IM; intestinal metaplasia, MAPS; management of epithelial precancerous conditions and lesions in the stomach*
